# Oral anticoagulants, time in therapeutic range and renal function over time in real-life patients with atrial fibrillation and chronic kidney disease

**DOI:** 10.1136/openhrt-2022-002043

**Published:** 2022-09-14

**Authors:** Gorav Batra, Angelo Modica, Henrik Renlund, Anders Larsson, Christina Christersson, Claes Held

**Affiliations:** 1 Department of Medical Sciences, Cardiology, Uppsala University, Uppsala, Sweden; 2 Uppsala Clinical Research Center, Uppsala, Sweden; 3 Pfizer AB, Sollentuna, Sweden; 4 Department of Medical Sciences, Clinical Chemistry, Uppsala University, Uppsala, Sweden

**Keywords:** atrial fibrillation, pharmacology, clinical, arrhythmias, cardiac

## Abstract

**Aims:**

To describe the use of warfarin and direct oral anticoagulants (DOACs) in patients with atrial fibrillation (AF) and chronic kidney disease (CKD), to evaluate changes in renal function over time and predictors of rapid decline, and to describe time in therapeutic range (TTR) and predictors of poor TTR among patients on warfarin.

**Methods and results:**

Using data from AuriculA, the Swedish oral anticoagulation registry, patients with AF on warfarin or DOAC were identified between 2013 and 2018 (N=6567). Estimated glomerular filtration rate (eGFR) was calculated and categorised into normal (≥90 mL/min/1.73 m^2^), mild CKD (60–89 mL/min/1.73 m^2^), moderate CKD (30–59 mL/min/1.73 m^2^), severe CKD (15–29 mL/min/1.73 m^2^) and end-stage CKD (<15 mL/min/1.73 m^2^)/dialysis. TTR was estimated using international normalised ratio (INR) measurements. Predictors of eGFR decline over time and of poor TTR were estimated using regression analysis. Between 2013 and 2018, use of DOAC increased from 9.2% to 89.3%, with a corresponding decline in warfarin. A similar trend was observed in patients with mild to moderate CKD, while DOAC over warfarin increased slower among patients with severe to end-stage CKD/dialysis. In patients treated with warfarin, the median TTR was 77.1%. Worse TTR was observed among patients with severe CKD (70.0%) and end-stage CKD/dialysis (67.5%). A gradual annual decline in eGFR was observed (−1.1 mL/min/1.73 m^2^), with a more rapid decline among patients with older age, female sex, diabetes mellitus and/or heart failure.

**Conclusion:**

In patients with AF, use of DOAC has steadily increased across different CKD stages, but not in patients with severe to end-stage CKD/dialysis despite these patients having poor INR control. Patients with AF have a gradual decline in renal function, with a more rapid decline among a subgroup of patients.

**WHAT IS ALREADY KNOWN ON THIS TOPIC**

- There are conflicting data on whether warfarin treatment is safe and effective in patients with chronic kidney disease (CKD), particularly in those with end-stage CKD.
- The absolute change in renal function over time and the predictors of rapid decline in renal function among patients treated with warfarin or direct oral anticoagulants (DOACs) are also unknown.

**WHAT THIS STUDY ADDS**

- Treatment with DOAC has increased drastically between 2013 and 2018, with a corresponding decrease in use of warfarin in patients with atrial fibrillation (AF).
- Similar trends were seen in patients with mild to moderate CKD, while use of DOAC in preference to warfarin increased considerably slower among patients with severe CKD and end-stage CKD/dialysis.
- In patients treated with warfarin, poor warfarin control with low time in therapeutic range was observed to a higher extent among those with severe CKD and end-stage CKD/dialysis.
- Also, there was a gradual decline in renal function over time in patients with AF, with a more rapid reduction among patients with older age, female sex, diabetes mellitus and/or heart failure.

**HOW THIS STUDY MIGHT AFFECT RESEARCH, PRACTICE OR POLICY**

- This study underlines the importance of close follow-up of renal function in selected patients at risk to allow timely adjustment of oral anticoagulant treatment in terms of drug of choice and correct dosage.
- For patients on warfarin with severe CKD and end-stage CKD/dialysis, close follow-up of international normalised ratio or change in oral anticoagulant strategy might be warranted.

## Introduction

Clinical practice guidelines recommend direct oral anticoagulants (DOACs) over warfarin for stroke prevention in patients with atrial fibrillation (AF) having risk factors for ischaemic stroke.^1 2^ This recommendation is mainly based on the favourable safety profile of DOACs, especially in terms of fewer intracranial bleedings compared with vitamin K antagonists, for example, warfarin.^3–6^ However, the risk of suffering from major bleeding due to treatment with oral anticoagulants (OAC) is highly associated with the individual patient risk profile.^3–6^ A relevant example is the increased risk of bleeding among patients with AF and chronic kidney disease (CKD), an increasingly common comorbidity owing in part to ageing population.^7^ Moreover, patients with AF and CKD have an inherent worse prognosis, not only in terms of more frequent bleeding events, but also due to higher risk of thromboembolic events and mortality, in comparison with the general AF populations.^8^

All DOACs are dependent on renal clearance to various degrees, causing a potential risk of excessive drug exposure and consequently a higher risk of associated bleeding in patients with CKD.^9^ For example, in a subgroup of patients with CKD in the ARISTOTLE (Apixaban for Reduction in Stroke and Other Thromboembolic Events in Atrial Fibrillation) trial, apixaban was superior to warfarin in preventing stroke or systemic embolism and bleeding events. There was an observation of a positive statistical interaction suggesting that patients with worsened renal function had a greater benefit with apixaban versus warfarin in terms of major bleeding.^10^ Also, observational studies with various other DOACs in patients with AF and CKD have suggested a favourable effect with DOACs compared with warfarin.^11^ Despite fixed daily dosages of DOACs, the dependency on renal clearance necessitates monitoring of renal function over time as it might gradually decrease.^9^ How often renal function should be monitored is unclear. For warfarin, there are conflicting observational data on whether treatment is safe and effective in patients with CKD, particularly in patients with end-stage CKD in whom there is a risk of calciphylaxis and suboptimal international normalised ratio (INR) control and time in therapeutic range (TTR).^12 13^ Also, it has been speculated that the combined increased risk of stroke and bleeding observed in patients with CKD and warfarin might be due to poor INR control.^12 14^

Despite reports suggesting favourable benefit to risk ratios with DOAC versus warfarin in patients with worsening renal function, the Swedish Medical Product Agency recommends warfarin over DOAC in patients with CKD and with estimated glomerular filtration rate (eGFR) below 25–30 mL/min/1.73 m^2^.^15^ The rationale behind this recommendation is the uncertainty with DOAC in patients with severe CKD, mainly due to the scarcity of data from randomised controlled trials (RCTs). However, this recommendation differs slightly from European clinical practice guidelines, which suggest that some DOACs might be used down to eGFR of 15 mL/min/1.73 m^2^.^1^

Currently, there are ongoing RCTs evaluating DOACs versus warfarin in patients with severe to end-stage CKD. While awaiting the results, it is important to understand how healthcare professionals weigh the benefits and risks and interpret the data in relation to guidelines when deciding which OAC to prescribe to patients with AF and CKD. Thus, the aims of the present real-world study were to illustrate (1) treatment patterns with warfarin and DOACs in patients with AF and CKD, (2) TTR in patients with AF and CKD treated with warfarin, (3) predictors of poor TTR in patients treated with warfarin, (4) changes in eGFR over time in patients treated with warfarin or DOACs, and (5) predictors of worsening eGFR in patients treated with warfarin or DOACs.

## Methods

### Study population

The study cohort was identified in the Swedish national quality register for AF and oral anticoagulation (AuriculA). AuriculA has over 150 000 active patients and is estimated to include approximately 50% of all patients with AF in Sweden.^16^ The registry encompasses data on, for example, indication for treatment with OACs, data on specific OACs and dosages, and information on INR in patients treated with warfarin. In this study, all patients living in the region of Uppsala, Sweden with a diagnosis of AF registered in AuriculA between 1 January 2013 and 31 December 2018 were included based on the availability of data on renal function. Patients aged <18 years and with mechanical heart valves or mitral stenosis were excluded, resulting in 6567 patients being included (online supplemental figure S1). Data on patient characteristics and pharmacy-dispensed medications (other than OAC) were obtained by linking AuriculA with the National Patient Register (NPR) and the Swedish Prescribed Drug Register. NPR, which has previously been shown to have high validity, is a mandatory registry for all inpatient and outpatient encounters in Sweden and includes diagnosis codes for patients seeking hospital care based on the International Statistical Classification of Diseases system (online supplemental table S1).^17^ Linking between AuriculA, NPR and the Swedish Prescribed Drug Register was approved and performed by the National Board of Health and Welfare in Sweden using the unique civic registration number available to all Swedish citizens. Data on renal function (creatinine) were obtained for the study cohort from laboratory databases and were linked to the registry by the National Board of Health and Welfare. Index date was the day of first treatment prescription for warfarin or DOAC during the study period.

10.1136/openhrt-2022-002043.supp1 Supplementary data

### Exposure and outcomes

Information about medical treatment with warfarin or DOAC and dosages was obtained from AuriculA. Information about eGFR was calculated based on serum creatinine concentrations using the Chronic Kidney Disease Epidemiology Collaboration equation.^18^ The serum creatinine measured closest within ±6 months to index date was used to calculate the baseline eGFR. All creatinine methods in the study were isotope dilution mass spectrometry (IDMS) calibrated. The equations used for eGFR were all adapted for IDMS-calibrated creatinine methods. For illustrative purposes, CKD was categorised into commonly used CKD stages as defined by the Kidney Disease: Improving Global Outcomes organisation: normal (eGFR ≥90 mL/min/1.73 m^2^), mildly decreased (eGFR 60–89 mL/min/1.73 m^2^), moderately decreased (eGFR 30–59 mL/min/1.73 m^2^), severely decreased (eGFR 15–29 mL/min/1.73 m^2^) and end-stage CKD (eGFR <15 mL/min/1.73 m^2^)/dialysis.^19^ Worsening eGFR over time was defined as a drop in eGFR ≥20% over a 1-year period based on its clinical relevance in previous studies.^20 21^ When analysing TTR in patients treated with warfarin, all available INR measurements during follow-up were used to estimate the median TTR. Linear interpolation was used to calculate the TTR of each patient and was presented as the percentage of time that the INRs were within the therapeutic range of between 2.0 and 3.0, using the Rosendaal method.^22^

### Statistics

Patient characteristics, risk factors, comorbidities and medical treatment were reported in a tabular format with median and IQR for continuous variables and with frequencies and percentages for categorical variables across the prespecified CKD strata. Statistical differences were reported using Kruskal-Wallis for continuous variables and using χ^2^ for categorical variables. Treatment strategies with warfarin and DOACs over years were illustrated using line graphs with proportional percentages. Multiple regression analysis was used to assess the association between CKD stage and TTR, with TTR being the dependent variable and baseline characteristics being the independent variables (age, sex, CKD stage, hypertension, diabetes mellitus, prior stroke/systemic embolism/transient ischaemic attack (TIA), prior myocardial infarction, prior revascularisation (percutaneous coronary intervention or coronary artery bypass graft surgery), heart failure, peripheral vascular disease, chronic obstructive pulmonary disease (COPD), cancer, prior major bleeding, and concomitant antiplatelet therapy (aspirin or P2Y_12_ inhibitor)). This analysis was performed on patients treated with warfarin and in whom serial INR measurements were available (n=3007, 45.8%). Temporal changes in eGFR from index date and onwards were illustrated using line graphs indicating median values with CIs. The association between baseline characteristics and the annual change in eGFR was examined using logistic regression analysis, where an annual drop in eGFR ≥20% was the response variable, with baseline characteristics being the independent variables (age, sex, CKD stage, hypertension, diabetes mellitus, prior stroke/systemic embolism/TIA, prior myocardial infarction, prior revascularisation, heart failure, peripheral vascular disease, COPD, cancer, prior major bleeding). This analysis was performed in patients with available serial measurements of eGFR (n=4055, 61.7%). All statistical analyses were performed using R V.4.1.1 (The R Foundation). Two-sided p value <0.05 was considered statistically significant.

## Results

### Baseline characteristics

**Figure 1:**
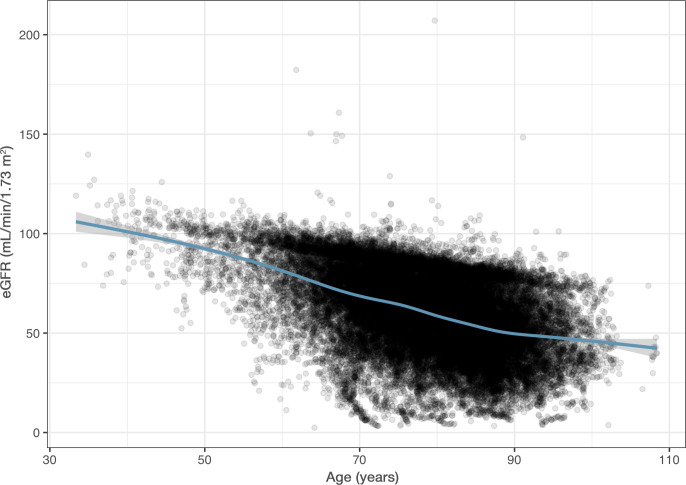
Estimated glomerular filtration rate (eGFR) at baseline in relation to age.

The median age of the population was 77.2 years and 42.3% were female. The baseline characteristics of all patients are presented by CKD stage (eGFR ≥90, 60–89, 30–59, 15–29 and <15/dialysis) in table 1. Patients with worsening CKD stage were older, often male and had more comorbidities, including hypertension, diabetes mellitus, prior stroke, systemic embolism, prior myocardial infarction, heart failure, peripheral vascular disease, prior major bleeding, and correspondingly higher CHA_2_DS_2_-VASc score (congestive heart failure, hypertension, age, diabetes mellitus, stroke/transient ischemic attack/thromboembolism history, vascular disease history and sex) (figure 1 and table 1).

**Table 1:** Baseline characteristics

Characteristics	eGFR ≥90 (n=618)	eGFR 60–89 (n=3775)	eGFR 30–59 (n=1965)	eGFR 15–29 (n=175)	eGFR <15/dialysis (n=34)
Demographics					
Age, years, median (IQR)	63.6 (57.4–68.7)	75.7 (69.7–82.2)	83 (76.9–88.2)	85.8 (78.8–91.0)	79.2 (75.3–84.3)
Sex, female, n (%)	461 (74.6)	2264 (60.0)	957 (48.7)	84 (48.0)	23 (67.6)
Medical history, n (%)					
Hypertension	294 (47.6)	1985 (52.6)	1371 (69.8)	132 (75.4)	31 (91.2)
Diabetes mellitus	104 (16.8)	549 (14.5)	390 (19.8)	58 (33.1)	12 (35.3)
Prior stroke	45 (7.3)	316 (8.4)	260 (13.2)	25 (14.3)	5 (14.7)
Prior TIA	15 (2.4)	176 (4.7)	103 (5.2)	14 (8.0)	3 (8.8)
Prior systemic embolism	2 (0.3)	22 (0.6)	29 (1.5)	7 (4.0)	1 (2.9)
Prior MI	34 (5.5)	290 (7.7)	214 (10.9)	35 (20.0)	5 (14.7)
Prior PCI or CABG	46 (7.4)	385 (10.2)	283 (14.4)	43 (24.6)	6 (17.6)
Heart failure	39 (6.3)	294 (7.8)	368 (18.7)	68 (38.9)	9 (26.5)
Peripheral vascular disease	17 (2.8)	155 (4.1)	112 (5.7)	22 (12.6)	5 (14.7)
COPD	38 (6.1)	226 (6.0)	143 (7.3)	19 (10.9)	2 (5.9)
Cancer (within 3 years)	29 (4.7)	188 (5.0)	132 (6.7)	12 (6.9)	4 (11.8)
Prior major bleeding	35 (5.7)	215 (5.7)	186 (9.5)	29 (16.6)	6 (17.6)
CHA_2_DS_2_-VASc, median (IQR)	1 (0–2)	2 (1–4)	4 (2–4)	4 (3–5)	4 (3–5)
Biochemical analyses, median (IQR)					
Number of eGFR measures	4 (2–6)	4 (2–7)	5 (2–9)	5 (2–10)	5 (2–8)
eGFR (mL/min/1.73 m^2^)	94.6 (92.2–98.5)	75.3 (68.0–82.1)	49.4 (42.1–55.5)	25.7 (22.4–28.2)	12.0 (10.2–12.9)
Number of INR measures (n=3602)	44 (19–73)	50 (24–78)	49 (21–79)	31 (13–62)	43 (12–86)
INR (g/L)	2.4 (2.1–2.8)	2.4 (2.1–2.8)	2.4 (2.1–2.8)	2.0 (2.1–2.8)	2.0 (2.1–2.9)
TTR (%) (n=3514)	73.7 (62.5–83.5)	78.6 (68.4–85.8)	76.3 (66.7–84.2)	70.0 (54.1–79.1)	67.3 (53.8–77.0)
TTR >70%, n (%)	183 (58.1)	1441 (72.4)	729 (68.5)	58 (49.6)	12 (42.9)
TTR >60%–70%, n (%)	65 (20.6)	281 (14.1)	165 (15.5)	23 (19.7)	5 (17.9)
TTR <60%, n (%)	67 (21.3)	267 (13.4)	171 (16.1)	36 (30.8)	11 (39.3)
Medication, n (%)					
Warfarin	331 (53.6)	2064 (54.7)	1113 (56.6)	126 (72.0)	32 (94.1)
DOAC	287 (46.4)	1708 (45.2)	851 (43.3)	49 (28.0)	2 (5.9)
Dabigatran etexilate	67 (10.8)	292 (7.7)	102 (5.2)	0 (0.0)	0 (0.0)
Rivaroxaban	27 (4.4)	202 (5.4)	103 (5.2)	1 (0.6)	1 (2.9)
Apixaban	193 (31.2)	1211 (32.1)	646 (32.9)	48 (27.4)	1 (2.9)
Edoxaban	0 (0.0)	3 (0.1)	0 (0.0)	0 (0.0)	0 (0.0)
Other OACs	0 (0.0)	3 (0.1)	1 (0.1)	0 (0.0)	0 (0.0)
Acetylsalicylic acid	121 (19.6)	985 (26.1)	632 (32.2)	77 (44.0)	16 (47.1)
P2Y_12_ inhibitor	14 (2.3)	132 (3.5)	75 (3.8)	11 (6.3)	2 (5.9)

Values are median (IQR) for continuous variables and n (%) for categorical variables.

eGFR levels are based on the Chronic Kidney Disease Epidemiology Collaboration equation.

CABG, coronary artery bypass grafting; CHA2DS2-VASc, congestive heart failure, hypertension, age, diabetes mellitus, stroke/transient ischemic attack/thromboembolism history, vascular disease history and sex; COPD, chronic obstructive pulmonary disease; DOAC, direct oral anticoagulant; eGFR, estimated glomerular filtration rate; INR, international normalised ratio; MI, myocardial infarction; OAC, oral anticoagulant; PCI, percutaneous coronary intervention; TIA, transient ischaemic attack; TTR, time in therapeutic range.

### Treatment patterns for OACs in relation to CKD stage

**Figure 2:**
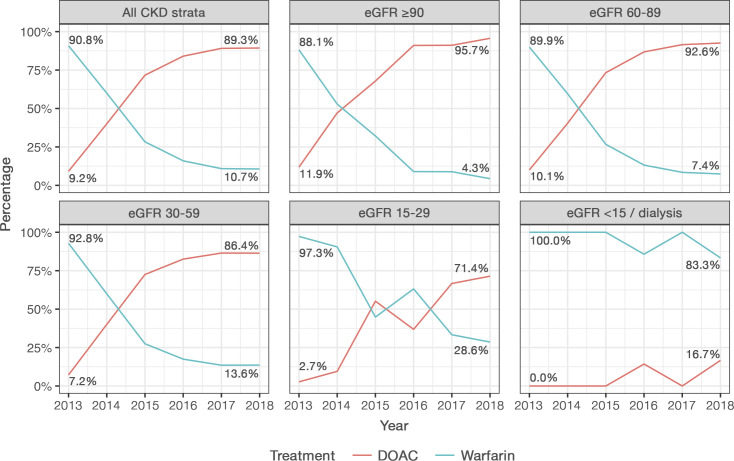
Treatment initiation with warfarin or DOAC between 2013 and 2018 in Uppsala County based on CKD stage. CKD, chronic kidney disease; DOAC, direct oral anticoagulant; eGFR, estimated glomerular filtration rate.

Between 2013 and 2018, the proportion of patients treated with warfarin decreased from 90.8% to 10.7%, while the proportion treated with DOAC increased from 9.2% to 89.3% (figure 2). When categorised into CKD stages, a similar trend was observed in patients with normal (eGFR ≥90), mildly decreased (eGFR 60–89) and moderately decreased renal function (eGFR 30–59). However, in patients with severely decreased renal function (eGFR 15–29), the change in number of patients treated with DOAC compared with warfarin was slower, while patients with end-stage CKD (eGFR <15) or on dialysis more often received warfarin than DOAC over time (figure 2).

### TTR in patients treated with warfarin across various CKD stages

**Figure 3:**
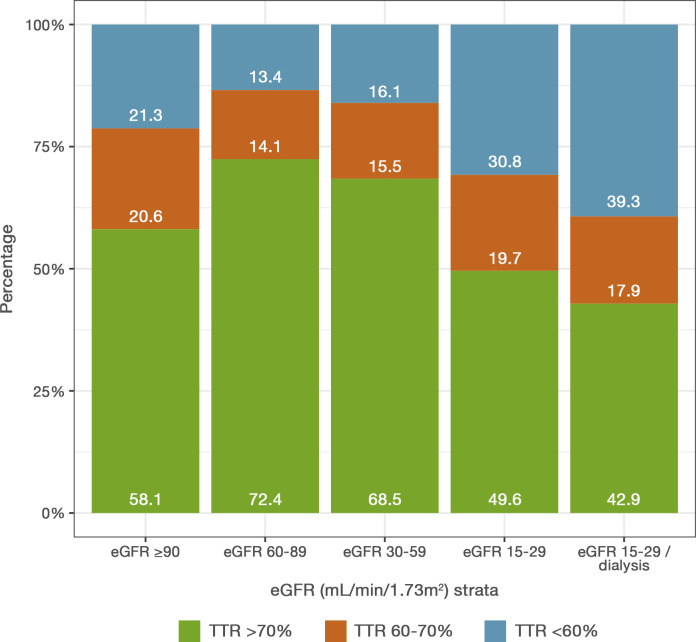
Proportion of patients in different TTR ranges across various chronic kidney disease stages. eGFR, estimated glomerular filtration rate; TTR, time in therapeutic range.

In total, 190 814 INR measurements (median of 49 measurements per patient) were available during a median follow-up of 1.7 years. The median TTR was 77.1% in patients treated with warfarin, with marginally poorer TTR observed among patients with worsening CKD stage (table 1). In patients with normal renal function, 58.1% of patients had a good TTR (≥70%). In patients with mild to moderately decreased renal function, an even higher proportion of patients had a good TTR (72.4% and 68.5%, respectively). A good TTR was to a lower extent observed among patients with severe CKD or end-stage CKD/dialysis (49.6% and 42.9%, respectively) (figure 3).

Clinical predictors associated with poor TTR are presented in table 2. In the fully adjusted multivariable model, factors independently associated with poor TTR included diabetes mellitus, heart failure, COPD and concomitant treatment with acetylsalicylic acid. There was a non-significant trend towards worsening TTR among patients with end-stage CKD/dialysis.

**Table 2:** Predictors of poor TTR

Characteristics	Coefficient (95% CI)	P value
CKD stages		
eGFR ≥90	Reference	
eGFR 60–89	3.9 (2.2 to 5.6)	<0.001
eGFR 30–59	3.0 (1.1 to 5.0)	0.002
eGFR 15–29	−0.9 (−4.0 to 2.2)	0.58
eGFR <15/dialysis	−5.5 (−11.0 to 0.1)	0.05
Age (/10 years)	0.4 (−0.1 to 1.0)	0.12
Sex, female	−0.7 (−1.6 to 0.2)	0.14
Hypertension	0.2 (−0.8 to 1.1)	0.70
Diabetes mellitus	−2.1 (−3.4 to −0.9)	<0.001
Prior stroke/TIA/systemic embolism	0.6 (−0.7 to 1.9)	0.34
Prior MI	0.7 (−1.3 to 2.6)	0.50
Prior PCI or CABG	0.2 (−1.5 to 1.9)	0.82
Heart failure	−3.5 (−4.9 to −2.2)	<0.001
Peripheral vascular disease	−1.8 (−3.9 to 0.3)	0.10
COPD	−6.3 (−8.2 to −4.5)	<0.001
Cancer (within 3 years)	−0.2 (−2.1 to 1.8)	0.86
Prior major bleeding	−1.6 (−3.4 to 0.2)	0.08
Acetylsalicylic acid	−1.5 (−2.6 to −0.4)	0.01
P2Y_12_ inhibitor	0.5 (−2.5 to 3.5)	0.74

CABG, coronary artery bypass grafting; CKD, chronic kidney disease; COPD, chronic obstructive pulmonary disease; eGFR, estimated glomerular filtration rate; MI, myocardial infarction; PCI, percutaneous coronary intervention; TIA, transient ischaemic attack; TTR, time in therapeutic range.

### Renal function (eGFR) over time

**Figure 4:**
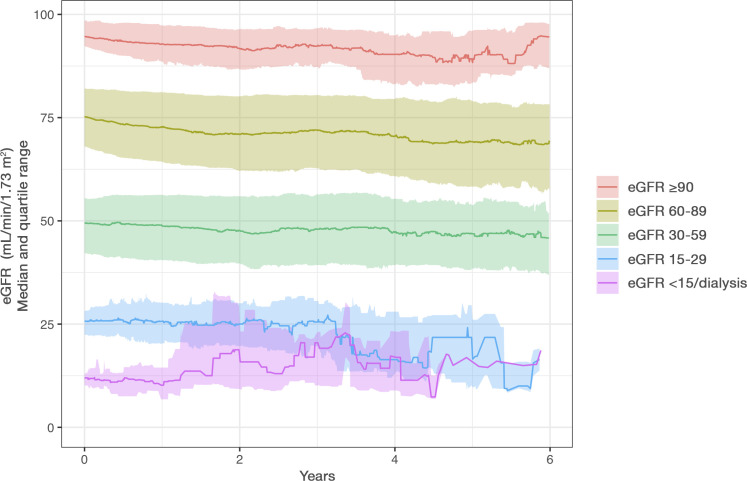
Median change in estimated glomerular filtration rate (eGFR) over time in patients treated with oral anticoagulants.

In total, 36 433 eGFR measurements (median of 4 measurements per patient) were available during follow-up. Serial eGFR measurements with at least two measurements over a 1-year period were available in 4055 (61.7%) patients included in this study. Among the subgroup of patients with serial measurements of eGFR, the median annual decline in eGFR was −1.1 (25th–75th percentile: −4.4 to 1.3) mL/min/1.73 m^2^, and a total of 1415 (34.9%) patients had a decline in eGFR ≥20% from baseline (figure 4 and online supplemental table S2). In patients treated with warfarin or DOAC, the median annual decline in eGFR was similar at −1.1 (−4.2 to 0.9) mL/min/1.73 m^2^ and −1.1 (−4.7 to 2.1) mL/min/1.73 m^2^, and a total of 955 (36.6%) and 460 (31.9%) patients had a decline in eGFR ≥20% from baseline, respectively (online supplemental table S2 and figure S2). In addition, among the 3331 patients with eGFR ≥50 at baseline, a total of 813 (24.4%) developed eGFR <50 (dose reduction criteria for dabigatran etexilate, rivaroxaban and edoxaban) during a median follow-up of 1.7 years. Similarly, among the 3945 patients with eGFR ≥30 at baseline, a total of 319 (8.1%) developed eGFR <30 (dose reduction criteria for apixaban) during follow-up (online supplemental table S2).

Worsening eGFR with an annual decline ≥20% from baseline was associated with several patient characteristics and comorbidities in the adjusted analysis, including age, female sex, diabetes mellitus and heart failure (table 3).

**Table 3:** Predictors of worsening renal function (≥20% decline in eGFR) over time

Characteristics	OR (95% CI)	P value
CKD stages		
eGFR ≥90	Reference	
eGFR 60–89	1.13 (0.79 to 1.61)	0.51
eGFR 30–59	1.05 (0.71 to 1.55)	0.82
eGFR 15–29	1.09 (0.61 to 1.97)	0.77
eGFR <15/dialysis	1.25 (0.39 to 3.97)	0.71
Age (/10 years)	1.49 (1.35 to 1.65)	<0.001
Sex, female	1.29 (1.10 to 1.52)	0.002
Hypertension	1.17 (0.98 to 1.39)	0.09
Diabetes mellitus	1.55 (1.27 to 1.90)	<0.001
Prior stroke/TIA/systemic embolism	0.75 (0.60 to 0.95)	0.02
Prior MI	1.01 (0.73 to 1.40)	0.93
Prior PCI or CABG	1.13 (0.85 to 1.49)	0.41
Heart failure	1.91 (1.53 to 2.39)	<0.001
Peripheral vascular disease	1.31 (0.93 to 1.83)	0.12
COPD	1.34 (0.99 to 1.81)	0.06
Cancer (within 3 years)	0.81 (0.57 to 1.16)	0.26
Prior major bleeding	1.21 (0.89 to 1.63)	0.23

CABG, coronary artery bypass grafting; CKD, chronic kidney disease; COPD, chronic obstructive pulmonary disease; eGFR, estimated glomerular filtration rate; MI, myocardial infarction; PCI, percutaneous coronary intervention; TIA, transient ischaemic attack.

## Discussion

We showed in this real-world evidence study that use of DOAC has increased between 2013 and 2018, with a corresponding decrease in warfarin in patients with AF. A similar trend was seen for patients with mild to moderate CKD, while use of DOAC in preference to warfarin increased considerably slower among patients with severe CKD and end-stage CKD/dialysis. In warfarin-treated patients, poor TTR (<70%) was observed to a higher degree among patients with severe CKD and end-stage CKD/dialysis. In addition, this study verified that there is a gradual decline in renal function over time in patients with AF, with a more rapid reduction among patients with older age, female sex, diabetes mellitus and heart failure.

CKD is a common finding among patients with AF and it is believed that CKD might contribute to the development and progression of AF.^23^ Both CKD and AF share common risk factors, including hypertension and diabetes mellitus, which might explain the mechanisms linking both diseases despite a causal pathway not being fully elucidated.^24^ Clinical practice guidelines recommend treatment with OACs, and preferably DOACs, of patients with AF and risk of ischaemic stroke.^1 2^ Furthermore, guidelines recommend assessment of thromboembolic and bleeding risk before initiating OAC therapy. However, current risk scores for prediction of thromboembolism and bleeding in patients with AF and CKD are inadequate as they have not been validated in patients with CKD and do not take into account the level of renal function impairment.^25^ For patients already on warfarin and with TTR <70%, switching to DOAC is recommended. Similar recommendations are given to patients with AF and mild to moderate CKD, where DOAC versus warfarin in the landmark DOAC trials showed similar efficacy and better safety profile.^1^ However, for patients with severe CKD or end-stage CKD/dialysis, data regarding the safety and efficacy with DOACs versus warfarin are scarce. Clinical practice guidelines recommend reduced dosage regimens for rivaroxaban, apixaban and edoxaban as feasible options to patients with severe CKD.^1^ For patients with end-stage CKD and dialysis, the question regarding anticoagulant therapy for stroke prevention is highly debated as observational data have indicated higher risk of bleeding and uncertainty about potential benefits with OAC for stroke prevention.^26^ For warfarin, there are also concerns regarding possible risk of calciphylaxis, vascular calcification, vertebral fractures and nephropathy.^13 27 28^ Also, the safety and efficacy of warfarin are limited by a narrow therapeutic window which requires stringent INR control between 2.0 and 3.0 and optimally a TTR >70%, which might be difficult to achieve in patients with CKD.^29^

In the present study, we found that treatment with DOACs steadily increased with time since their introduction, with a concurrent decline in warfarin. This trend was observed in patients with mild to moderate CKD, but not in patients with severe CKD or end-stage CKD/dialysis. This is in accordance with current recommendations provided in guidelines and is similar to changes seen across other European countries.^23 30^ In our study, the median TTR was high as observed in other Swedish studies on warfarin.^12^ However, for patients treated with warfarin and with severe to end-stage CKD or dialysis, the proportion of those with adequate TTR was low, with only 49.6% and 42.9% of patients achieving TTR >70%, respectively. Furthermore, there was a trend towards end-stage CKD/dialysis being an independent predictor of poor TTR; however, this finding was not statistically significant probably due to the small sample size. Similar observations have previously been made in other studies showing that patients with CKD more often have supratherapeutic INRs and subsequently higher risk of major bleeding.^31^ The underlying mechanism linking CKD and poor INR control is unknown, but it has been speculated that renal dysfunction might reduce clearance of warfarin and that advanced CKD more often triggers therapy discontinuation and treatment with other drugs/interventions, which might alter warfarin concentrations.^32^ These findings have implications for warfarin treatment in patients with severe to end-stage CKD or dialysis and might be mitigated by more frequent and systematic INR sampling or by replacing warfarin with DOACs as they might have a supporting role in this situation, especially in patients with eGFR >15 mL/min/1.73 m^2^.

Another important finding in this study is that patients with AF have a gradual decline in renal function over time. These findings are in accordance with prior observations made in patients with AF.^20 33^ In the present study, the median annual decline in eGFR was −1.1 mL/min/1.73 m^2^, with a more pronounced decline observed among patients with older age, female sex, diabetes mellitus and heart failure. Thus, in patients with normal renal function and with no risk factors, there seems to be only a modest need for close monitoring of renal function. However, for patients with poor renal function at treatment initiation or for patients with the above risk factors for rapid decline in renal function, more frequent monitoring of renal function might be advisable. For patients treated with DOACs, which are all partially eliminated by the kidney, frequent monitoring of renal function might be justified in those with risk factors for rapid decline in renal function or for patients where dose reduction or treatment discontinuation might be warranted due to renal limitations. As an example, the results showed that a quarter of the patients with an eGFR above 50 mL/min/1.73 m^2^ had an eGFR below 50 mL/min/1.73 m^2^ after a median follow-up period of 1.7 years, a cut-off point where most of the DOACs need to be dose-adjusted.

The present study has several strengths and some limitations that merit acknowledgement. The key strength is the inclusion of consecutive real-life patients with AF with no loss to follow-up. Furthermore, the good availability of measurements of creatinine-based eGFR and INR and, in many patients, serial measurements of eGFR and INR are also strengths. However, for serial measurements of eGFR, only 61.7% of patients had two or more measurements available over a 1-year period, which is a limitation to parts of this study. The inclusion of patients with complete information on baseline characteristics and antithrombotic treatment provided opportunities to study the associations between renal function and TTR and time-dependent decline in renal function over time. A limitation is that we only had access to renal function covering patients in one region of Sweden, which might not be representative of nationwide estimates. Another limitation is that we did not have access to the INR measurements made using home monitoring devices. However, the study population had a median of 44 INR measurements per patient during the entire follow-up and the INR control in this cohort is likely to be closer to daily clinical practice than in clinical trials. Finally, we only accounted for comorbidities and medications available at baseline when assessing the associations between renal function, TTR and decline in renal function over time and thus residual confounders might be present.

## Conclusions

In real-life patients with AF, treatment with DOAC over warfarin has dramatically increased during the last years across normal and moderate levels of CKD. However, this trend was not observed among patients with severe to end-stage CKD/dialysis despite these patients having poor INR control. A gradual decline in renal function was observed among patients with AF over time, with a more rapid decline observed among those with older age, female sex, diabetes mellitus and/or heart failure. A close follow-up of patients at risk might be crucial to timely adjust OAC treatment in terms of drug of choice and correct dosage.

## Data Availability

Data may be obtained from a third party and are not publicly available. AuriculA does not allow individual data sharing to third parties. Access to aggregated data might be granted following review by the AuriculA steering committee. Such requests can be submitted to the AuriculA steering committee for consideration.
