# Supplementary material for: Oral anticoagulants, time in therapeutic range and renal function over time in real-life patients with atrial fibrillation and chronic kidney disease
Source: Open Heart. 2022 Sep 14;9(2):e002043. doi: 10.1136/openhrt-2022-002043 (PMC9476150; doi:10.1136/openhrt-2022-002043)
Supplement: Supplementary data [file openhrt-2022-002043supp001.pdf]

Supplementary material

Oral anticoagulants, time in therapeutic range, and renal function over time in real-life patients with atrial fibrillation and chronic kidney disease

Gorav Batra (MD, PhD), Angelo Modica (MD, PhD),
Henrik Renlund (MSc), Anders Larsson (MD, PhD),
Christina Christersson (MD, PhD), Claes Held (MD, PhD)

Batra G, *et al. Open Heart* 2022; 9:e002043. doi: 10.1136/openhrt-2022-002043

Supplementary Table S1. The International Code of Disease, tenth revision (ICD-10) and the Anatomical Therapeutic Chemical (ATC) codes applied to identify comorbidities and comedication

Variable	Data source	ICD-10 / ATC code
Age	AuriculA	-
Sex	AuriculA	-
Hypertension	NPR	I10, I11, I12, I13, I15
Diabetes mellitus	NPR	E10, E11, E12, E13, E14
Prior stroke	NPR	I60, I61, I63, I64
Prior TIA	NPR	G45
Prior systemic embolism	NPR	I74
Prior MI	NPR	I21, I22, I23, I252
Prior PCI or CABG	NPR	Z951, Z955
Heart failure	NPR	I42, I50, I110, I255, I130, I132, K761
Peripheral vascular disease	NPR	I70, I71, I72, I73
COPD	NPR	J43, J44
Cancer (within 3 years)	NPR	C
Prior major bleeding	NPR	I60, I61, I62, S064, S065, S066, K226, K250, K252, K254, K256, K260, K262, K264, K266, K270, K272, K274, K276, K280, K282, K284, K286, K290, K625, K661, K920, K921, K922, I850, I983, N02, R319, N939, N950, N501A, H113, H313, H356, H431, H450, H922, I312, J942, M250, R04, R58, T810, D500, D629, T792
CHA_2DS_2-VASc score	AuriculA, NPR	1 point each for: heart failure, hypertension, age 65- 74 years, diabetes, vascular disease, female sex; 2 points each for 75 years, thromboembolism.
Creatinine/eGFR	AuriculA	-
INR	AuriculA	-
Warfarin	AuriculA	-
DOAC	AuriculA	-
Dabigatran etexilate	AuriculA	-
Rivaroxaban	AuriculA	-
Apixaban	AuriculA	-
Edoxaban	AuriculA	-
Other OAC	AuriculA	-
Acetylsalicylic acid	Prescribed Drug Register	B01AC06
$P2Y_{12}$ inhibitor	Prescribed Drug Register	B01AC04, B01AC05, B01AC22, B01AC24

eGFR levels are based on the Chronic Kidney Disease Epidemiology Collaboration (CKD-EPI) equation.

Abbreviations: CABG, coronary artery bypass grafting; COPD, chronic obstructive pulmonary disease; DOAC, direct oral anticoagulants; eGFR, estimated glomerular filtration rate; INR, international normalised ratio; MI, myocardial infarction; NPR, National Patient Register; OAC, oral anticoagulants; PCI, percutaneous coronary intervention; TIA, transient ischaemic attack; TTR, time in therapeutic range.

Supplementary Figure S1. Consort diagram illustrating inclusion and exclusion of patients

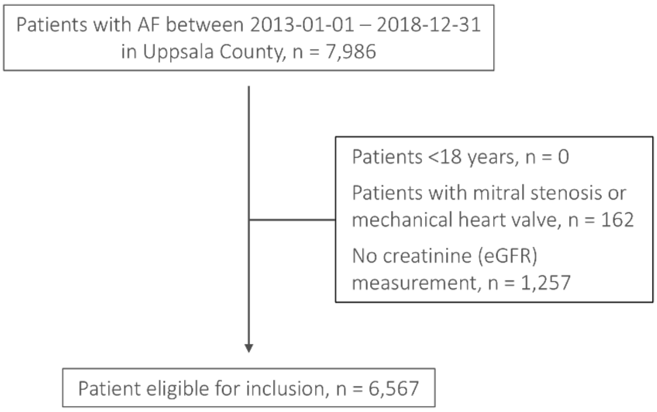

Supplementary Table S2. CKD stage at baseline (columns) and at end of follow-up (rows)

Baseline/<br>End follow-up	eGFR ≥90<br>(n = 364)	eGFR 60-89<br>(n = 2,384)	eGFR 30-59<br>(n =1,197)	eGFR 15-29<br>(n = 94)	eGFR <15/<br>dialysis<br>(n = 16)
**eGFR ≥90**	201 (55.2)	87 (3.7)	2 (0.2)	0 (0.0)	0 (0.0)
**eGFR 60-89**	152 (41.8)	1712 (71.8)	175 (14.6)	3 (3.2)	0 (0.0)
**eGFR 30-59**	11 (3.0)	561 (23.5)	881 (73.6)	24 (25.5)	4 (25.0)
**eGFR 15-29**	0 (0.0)	21 (0.9)	132 (11.0)	48 (51.1)	3 (18.8)
**eGFR <15/dialysis**	0 (0.0)	3 (0.1)	7 (0.6)	19 (20.2)	9 (56.3)

eGFR levels are based on the Chronic Kidney Disease Epidemiology Collaboration (CKD-EPI) equation.

Abbreviations: eGFR, estimated glomerular filtration rate

Supplementary Figure S2. Median change in eGFR over time in patients treated with (A) warfarin and (B) DOAC

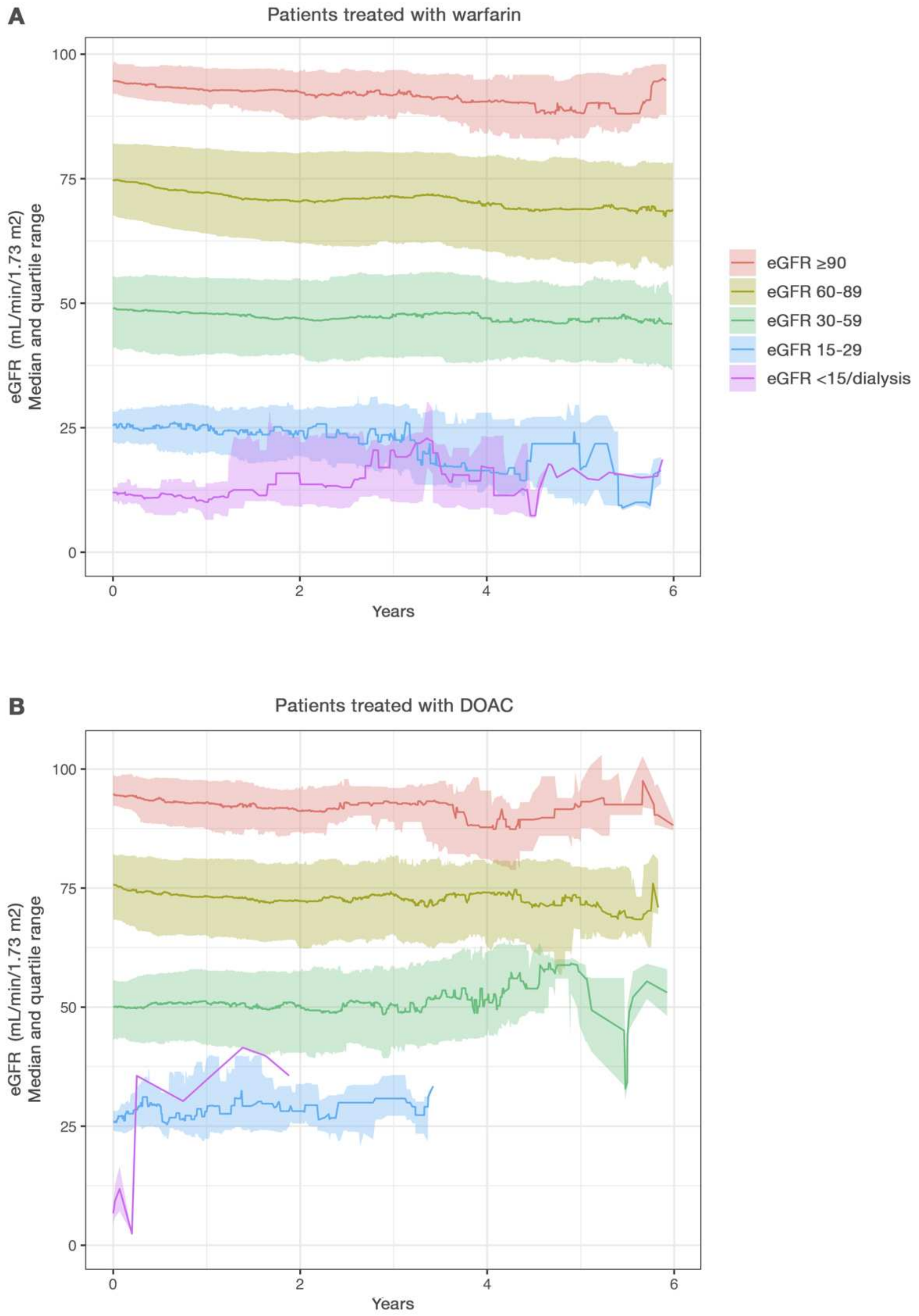

Batra G, *et al. Open Heart* 2022; 9:e002043. doi: 10.1136/openhrt-2022-002043
